# *Notes from the Field:* *Brucella abortus* Vaccine Strain RB51 Infection and Exposures Associated with Raw Milk Consumption — Wise County, Texas, 2017

**DOI:** 10.15585/mmwr.mm6709a4

**Published:** 2018-03-09

**Authors:** Caitlin M. Cossaboom, Grishma A. Kharod, Johanna S. Salzer, Rebekah V. Tiller, Lindsay P. Campbell, Karen Wu, María E. Negrón, Naomi Ayala, Nicole Evert, Jill Radowicz, Jennifer Shuford, Shelley Stonecipher

**Affiliations:** ^1^Epidemic Intelligence Service, CDC; ^2^Division of High Consequence Pathogens and Pathology, National Center for Emerging Zoonotic Infectious Diseases, CDC; ^3^Texas Department of State Health Services, Arlington, Texas; ^4^Texas Department of State Health Services, Austin, Texas.

In July 2017, the Texas Department of State Health Services (DSHS) Region 2/3 office reported a human case of brucellosis associated with the consumption of raw (unpasteurized) cow’s milk purchased from a dairy in Paradise, Texas. CDC’s Bacterial Special Pathogens Branch (BSPB) confirmed the isolate as *Brucella abortus* vaccine strain RB51 (RB51).

Brucellosis is a zoonotic bacterial disease that affects humans and many animal species. In humans, the disease is characterized by fever and nonspecific influenza-like symptoms that frequently include myalgia, arthralgia, and night sweats. Without appropriate treatment, brucellosis can become chronic, and life-threatening complications can arise. Human brucellosis transmitted by cattle was once common in the United States. Control strategies have focused on elimination of brucellosis through vaccination and surveillance of cattle herds, in addition to milk pasteurization. Because of these measures, domestically acquired human cases are now rare (*1**).*

RB51, a live-attenuated vaccine used to prevent *B. abortus* infection in cattle, has been documented to cause human disease, most commonly through occupational exposures such as needle sticks (*2*). Importantly, unlike wild strains of *B. abortus,* RB51 does not stimulate an antibody response detectable by routine serological assays, requiring culture for confirmation. Additionally, RB51 is resistant to rifampin, a common treatment choice for human brucellosis (*2**,**3*). This case represents the first documented instance of human brucellosis caused by RB51 through consumption of raw milk acquired in the United States.

Following isolation of RB51 from the patient’s blood, bulk milk tank samples from the farm tested positive for RB51 by polymerase chain reaction and bacterial culture. Culture of individual milk samples from all 43 cows in the herd identified two RB51 culture-positive cows. Subsequent whole genome sequencing indicated genetic relatedness between the cow and human isolate.

In Texas, farm sales of raw milk products to the public are legal with a “Grade ‘A’ Raw for Retail” license, regulated by the DSHS Milk and Dairy Group. By the end of August, through correspondence with the dairy, DSHS had identified approximately 800 persons who might have visited the farm during June 1–August 7. On September 1, Texas DSHS and BSPB began notification calls to these households, recommending that all exposed persons (i.e., those who consumed raw milk products from the farm during June 1–August 7) seek medical attention and begin 3 weeks of postexposure prophylaxis, even if asymptomatic (*4*).

Contact information was available for 582 households. The notification was issued successfully to 397 (68.2%) households. Among these notified households, 324 (81.6%) identified at least one exposed household member. Contacted persons referred 34 additional potentially exposed households, including households from seven other states.* A nationwide press release and Health Alert Network Health Advisory were issued in September to facilitate further identification of exposed persons (*5*). 

To date, there are no other confirmed cases associated with this investigation. CDC and Texas DSHS continue measures to increase awareness among health care providers and the public regarding unique challenges associated with treatment and diagnosis of RB51 in humans and the risks of consuming raw milk.

## Footnotes

* Alabama, Arkansas, California, North Dakota, Ohio, Oklahoma, and Tennessee.
